# Silent Storm: Advanced Heart Failure in a Young Female Patient With Systemic Lupus Erythematosus

**DOI:** 10.7759/cureus.98076

**Published:** 2025-11-29

**Authors:** Chris Sani, Godslove Bonnah, Dea Thomas, Victor Freitas De Souza, Tiffany Li, Ahmad Jallad

**Affiliations:** 1 Internal Medicine, State University of New York Downstate Health Sciences University, Brooklyn, USA; 2 Cardiology, State University of New York Downstate Health Sciences University, Brooklyn, USA; 3 Cardiology/Electrophysiology, Staten Island University Hospital, New York, USA; 4 Cardiology/Electrophysiology, State University of New York Downstate Health Sciences University, Brooklyn, USA

**Keywords:** end-stage renal disease, heart failure with reduced ejection fraction, lupus myocarditis, secondary pneumothorax, sle and lupus nephritis, systemic lupus erythematosus disease

## Abstract

Systemic lupus erythematosus is an autoimmune disorder involving autoantibody production and immune complex-mediated damage of multiple organ systems, including the heart. The complex systemic manifestations of lupus and late detection of cardiovascular involvement make it difficult to isolate a singular mechanism that causes heart failure. We present a case of a young female patient presenting with systemic lupus nephritis and Stage 4 lupus nephritis found to have advanced heart failure and associated complications. This case highlights a young patient with lupus who developed heart failure likely due to myocarditis, underscoring the need for early cardiovascular monitoring and targeted research to improve outcomes in this high-risk population. Lupus leading to heart failure is likely caused by a multifaceted approach; further studies are needed to tailor treatment strategies for these patients that can reduce the risk of heart failure.

## Introduction

Systemic lupus erythematosus (SLE) is a multiorgan system autoimmune connective tissue disorder characterized by widespread inflammation and immune dysregulation. This leads to a loss of self-tolerance due to B cell dysregulation, T cell abnormalities, aberrant activation of immune cells, and dysregulated cytokine production. Cardiovascular involvement is a major contributor to morbidity and mortality in SLE, with patients facing up to a 4.6-fold higher risk of developing heart failure (HF) compared to the general population [1]. Meanwhile, those who develop HF in SLE are at an increased rate of mortality with a hazard ratio of 1.5 compared to those who do not develop HF [2]. HF in SLE is commonly associated with myocarditis, which may present with dyspnea, chest pain, nausea, vomiting, cardiomegaly, tachycardia, or palpitations [3]. Myocarditis can progress to dilated cardiomyopathy and life-threatening HF with acute exacerbations of SLE. A comprehensive review of case reports revealed only 10 instances of SLE initially manifesting as dilated cardiomyopathy, with most patients being young women of childbearing age [4]. We present the case of a young female patient with SLE and Stage 4 lupus nephritis presenting with advanced manifestations of myocarditis and HF. The intensive management of her symptoms underscores the importance of early cardiovascular surveillance and intervention to mitigate the adverse cardiac complications of SLE in this high-risk population.

## Case presentation

A 21-year-old female patient from Trinidad with past medical history of SLE on chronic prednisolone and hydroxychloroquine therapy, HF with reduced ejection fraction (HFrEF) with a last known ejection fraction (EF) of 10%, end-stage renal disease (ESRD) on hemodialysis (HD) for five days, and seizure disorder managed with carbamazepine presents with progressively worsening shortness of breath. She had dyspnea even with minimal activity such as repositioning in bed. She had missed HD for two days prior to presentation due to travel and had a recent seizure episode three weeks earlier. Notably, she was previously advised to undergo pacemaker/defibrillator placement in Trinidad. On arrival, vital signs revealed a heart rate of 115 beats per minute, blood pressure of 138/105 mmHg, and a respiratory rate of 30 breaths per minute. Laboratory investigations were significant for elevated blood urea nitrogen (BUN) (53 mg/dL), creatinine (8.4 mg/dL), B-type natriuretic peptide (BNP) (754 pg/mL), glomerular filtration rate (GFR) (6 mL/min), and anemia (hemoglobin 7.7 g/dL) (Table 1). Chest radiograph demonstrated bilateral pleural effusions.

**Table 1:** Laboratory course during hospital events BUN: blood urea nitrogen; ICD: implantable cardioverter-defibrillator

Parameter (reference range)	Admission day	Day before ICD	Post 1st arrest	8 h post 1st arrest/3 h pre 2nd arrest	1 h post 2nd arrest	Post dialysis
Sodium (136–145 mmol/L)	134	136	136	134	140	136
Potassium (3.5–5.1 mmol/L)	4.0	3.6	4.5	7.3	5.7	4.7
BUN (7–25 mg/dL)	53	37	88	92	93	39
Creatinine (0.6–1.2 mg/dL)	8.4	4.7	11.0	11.4	11.3	6.5
pH (7.35–7.45)	7.39	–	7.01	7.35	7.37	–
Lactic acid (0.5–2.2 mmol/L)	0.9	–	–	0.8	1.8	–
WBC (3.50–10.0 K/µL)	6.0	7.23	10.18	15.1	17.99	12.2
Hemoglobin (12.0–16.0 g/dL)	7.7	9.0	9.5	8.5	9.3	7.7

**Figure 1:**
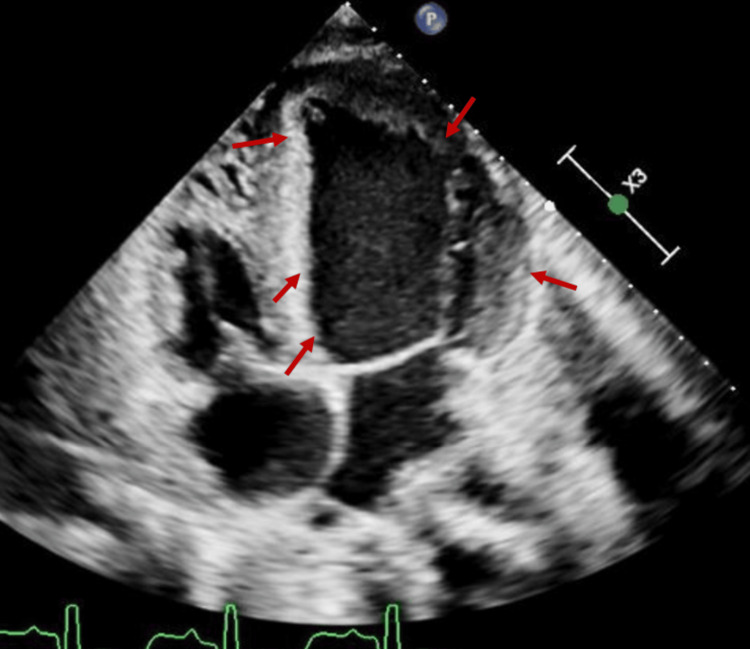
Apical view of a transthoracic echocardiogram showing a dilated left ventricle (red arrows)

The patient was admitted, and nephrology was consulted for emergent HD. She underwent dialysis on a hospital schedule of Tuesday, Thursday, and Saturday. A formal transthoracic echocardiogram performed two days post-admission showed a severely reduced EF of 22%, a dilated left ventricle (LV) (Figure 1) with severe diffuse hypokinesis, mildly reduced right ventricular (RV) systolic function, mild calcification of subvalvular apparatus trabeculations, dilated left atrium, and mild to moderate mitral and tricuspid regurgitation. Cardiology was consulted to evaluate the patient for potential implantable cardioverter-defibrillator (ICD) placement. Given her significant cardiac dysfunction, an ischemic workup was pursued. Coronary computed tomography (CT) angiography was recommended as coronary catheterization was deemed high-risk by the interventional cardiology team, but was unsuccessful due to an uncontrolled heart rate. Subsequently, a nuclear stress test was performed, which showed diffuse hypokinesis prominent in the inferior wall without a reversible defect. A transesophageal echocardiogram (TEE) excluded intracardiac thrombi, clearing the patient for ICD placement. However, during the ICD placement procedure, the patient experienced a generalized seizure followed by cardiac arrest with pulseless electrical activity. The patient was intubated; six cycles of chest compressions were completed with the administration of three doses of epinephrine, one dose of sodium bicarbonate, and one dose of calcium. Return of spontaneous circulation (ROSC) was achieved within 15 minutes, and the patient was taken to the Medical Intensive Care Unit (MICU).

**Figure 2:**
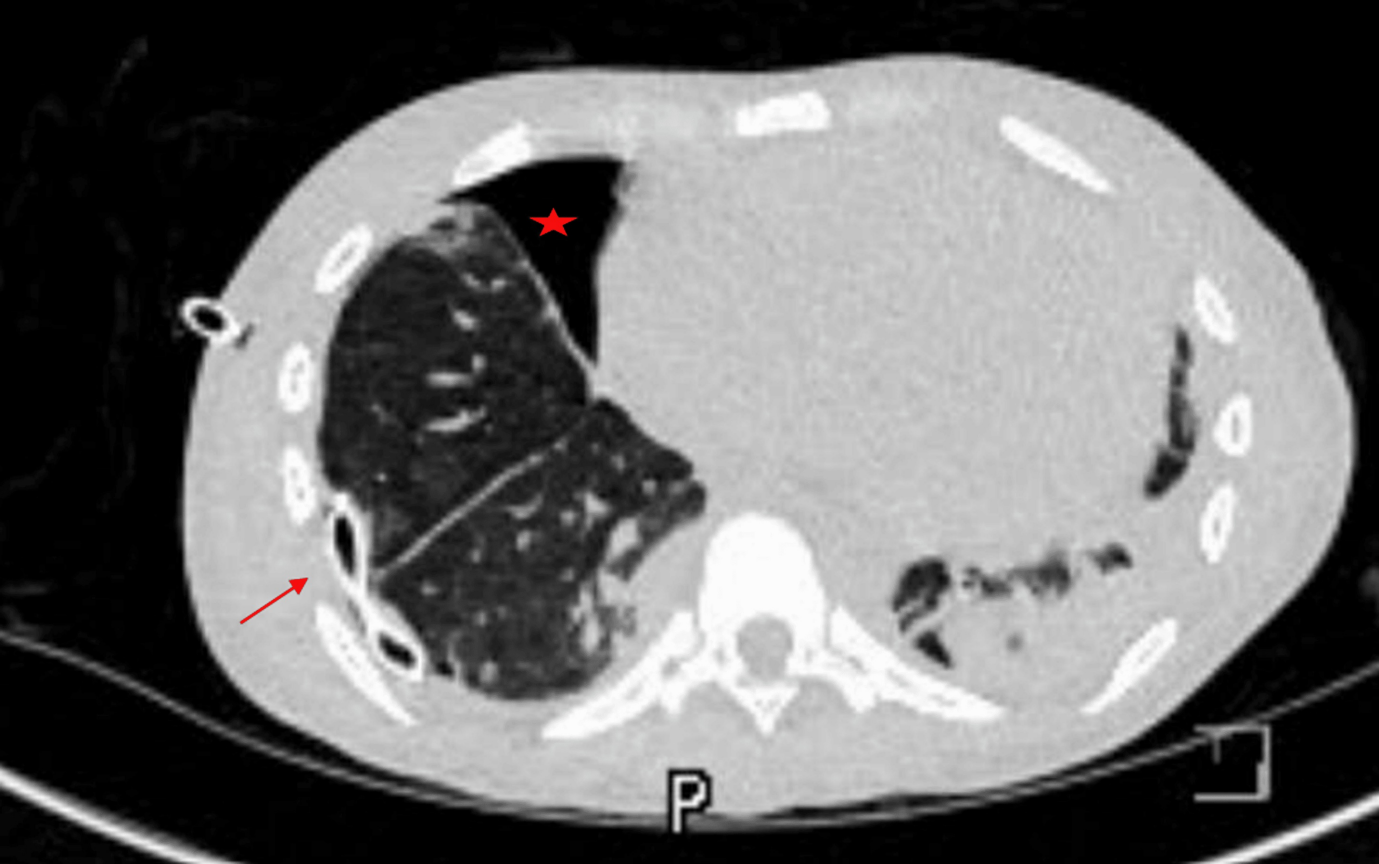
CT chest: enlarged heart, pneumothorax (red star) with chest tube (red arrow) in place CT: computed tomography

In the MICU, patients were started on midazolam and propofol drip, but hours later, the patient had another cardiac arrest due to hyperkalemia (Table 1). The patient was given two shocks for ventricular tachycardia and completed five rounds of CPR, at which ROSC was attained. Hyperkalemia treatment was initiated, and nephrology was consulted for emergent dialysis. The patient was initiated on levetiracetam for seizure prophylaxis and restarted on prednisone and hydroxychloroquine for SLE. The next day, an x-ray was obtained and demonstrated right-sided pneumothorax. Surgery was consulted, and a chest tube was placed; a CT scan was obtained, which showed that the chest tube was in place with hydropneumothorax (Figure 2), and repeat x-rays showed a stable pneumothorax. The patient required pressors but was later weaned off and was extubated to bilevel positive airway pressure (BIPAP). The patient was started on goal-directed medical therapy for HF, including carvedilol and sacubitril-valsartan.

Cardiology revisited discussions regarding ICD placement. However, the patient was apprehensive and declined further intervention. General surgery and pulmonology recommended transferring to a tertiary care facility with cardiothoracic surgery capabilities for further evaluation. The patient was transferred to an outside institution, where the chest tube was ultimately removed. She was discharged home with a wearable defibrillator and continues outpatient follow-up with the renal clinic for HD management.

## Discussion

SLE is a chronic inflammatory condition that affects the cardiovascular system and significantly increases cardiovascular risk. Patients with SLE have a significantly increased incidence of HF, with a relative risk of 4.6 (95% CI 4.3 to 4.9) when compared to the general population [1]. The development of HF in SLE patients may be because of several pathophysiological mechanisms including myocarditis, cardiomyopathy, or valvular heart disease as well as genetic predisposition [3]. Another hidden factor is the low socioeconomic status that primarily affects non-Hispanic Blacks and Hispanic populations, which is now a known etiology in worsening heart conditions [5]. Our 21-year-old patient demonstrates several possible pathophysiological mechanisms and clinical manifestations of SLE’s impact on the heart.

The main causative mechanism behind our patient’s HF is likely underlying myocarditis. Myocarditis is considered a rare manifestation of SLE, as it is seen in 5% to 10% of patients but has potentially fatal ramifications [6]. Post-mortem studies demonstrate that 50% to 80% of SLE patients have myocardial involvement, suggesting that subclinical cardiac inflammation remains unrecognized in a majority of SLE patients [7]. The pathophysiology of myocarditis is thought to be due to autoantibody formation, especially anti-SSA/Ro, aPL, and immune complex-mediated interstitial inflammation leading to secondary myocyte injury. Endomyocardial biopsies and post-mortem histology typically reveal interstitial fibrosis, inflammatory infiltrates, and myocyte hypertrophy. Studies have shown that LVEF reduction in patients with myocarditis occurs in about 25% of patients [8]. Most patients with SLE and myocarditis have preserved EF. In our patient, the echocardiogram revealed a moderately dilated LV and severely decreased EF of 20%. Additionally, interstitial inflammation is less prominent in patients undergoing corticosteroid therapy [9]. Corticosteroid therapy can lead to LV hypertrophy [3]. Endomyocardial biopsies, the gold standard for diagnosing myocardial involvement in inflammatory conditions like SLE, are rarely performed in practice due to their invasive nature. While cardiovascular magnetic resonance (CMR) is the gold standard for noninvasive myocarditis diagnosis in SLE and PET (especially fluorodeoxyglucose (FDG)-PET) can serve as a complementary modality for assessing active inflammation, neither was available at the primary facility.

Another major risk factor for HF in patients with SLE is coronary artery disease (CAD). Our patient underwent radionuclide myocardial perfusion imaging using regadenoson for ischemic evaluation, which revealed diffuse hypokinesis more pronounced in the inferior wall with a small reversible segment but without any fixed defects or EKG findings of ischemia. These findings are of low suspicion for ischemic heart disease, which is in keeping with existing literature, as only 21% of HF cases in SLE are attributable to CAD [10]. Additionally, long-term cohort studies have found that atherosclerotic events are more prevalent eight to nine years after the diagnosis of SLE in older (mean age of 54, p < 0.0001) male patients with comorbid hypertension, smoking, and obesity [10]. Although our patient does not fall into this demographic, CAD remains a possible mechanism, as she was diagnosed with HF eight years after the diagnosis of SLE, and her comorbid ESRD serves as an independent risk factor.

Our patient had several end-organ manifestations of SLE, including lupus nephritis with ESRD, which required dialysis five days per week via a left upper extremity arteriovenous fistula. Recent studies have shown that patients with SLE and lupus nephritis have an increased risk of cardiovascular risk factors including type 2 diabetes mellitus (T2DM), hypertension, and hyperlipidemia compared to those with SLE without concurrent lupus nephritis [2]. The presence of these risk factors further increases the likelihood of developing HF in these patients. Finally, SLE is associated with a form of endocarditis called marantic endocarditis due to vegetations leading to significant valve dysfunction leading to volume overload and HF [11]. While there were some calcifications seen in the subvalvular apparatus in our patient, there were no severe valvular pathologies seen on imaging. However, these cardiovascular risk factors are amenable to treatment, and adequately controlling them can modulate the risk of cardiovascular mortality and morbidity in these patients.

Current literature indicates that the drugs used in the management of SLE can either be cardioprotective or a dose-dependent risk factor that increases the probability of developing HF. The cardioprotective drugs mostly consist of immunosuppressive therapy like hydroxychloroquine, which inhibits cardiac inflammation [12]. There is limited data on the association between other drugs like mycophenolate mofetil, cyclophosphamide, and azathioprine therapy with the development of HF [2]. Biological disease-modifying antirheumatic drugs (bDMARDs) like rituximab and belimumab, which are used mainly in the management of rheumatoid arthritis (RA), have been shown to potentially reduce the risk of developing HF, but there is limited research on their use in SLE [13]. The use of steroids, which is a mainstay treatment option for SLE exacerbation and complications, is associated with an increased cardiovascular disease risk and the probability of developing HF [14,15].

The limitations that we have encountered with our case include the fact that the diagnosis of SLE was made in the patient’s home country, Trinidad, and therefore, we are not privy to the initial treatment interventions the patient received. We are also not aware if the patient was monitored for HF in the initial stages of diagnosis of SLE.

## Conclusions

Our patient illustrates a rare and severe manifestation of SLE, particularly affecting a young individual of color. The early detection of cardiac problems in SLE patients can be a difficult task, given the complex pathophysiology of SLE. Furthermore, HF remains a common yet understudied complication of SLE. Further research is needed to thoroughly study the relationship between SLE and HF and to develop management and monitoring strategies to curtail the effects of their coexistence on the health of individuals, especially titrating the research to younger patients with SLE and those of color as to prevent severe complications like those seen with this patient. There is a need for a streamlined approach to investigating patients with SLE for HF and related cardiac manifestations, and the immediate initiation of treatment when it is identified.
